# Supplementary material for: A comprehensive mechanosensory connectome reveals a somatotopically organized neural circuit architecture controlling stimulus-aimed grooming of the Drosophila head
Source: eLife. 2026 Jun 8;14:RP108044. doi: 10.7554/eLife.108044 (PMC13246004; doi:10.7554/eLife.108044)
Supplement: Peer review history

# Editor report: eLife Assessment

**DOI:** 10.7554/eLife.108044.3.sa0

**Authors:** Sonia Q Sen

**Affiliations:** Tata Institute for Genetics and Society India

**Keywords:** Convincing, Important

This **important** study provides a comprehensive map of how touch-sensitive neurons in the fly head connect to downstream circuits, revealing parallel pathways that preserve spatial organization and identifying a developmentally defined circuit linking sensory input to grooming behavior. The evidence is **convincing**, with detailed anatomical reconstruction and quantitative analysis supporting the main claims, while the link to behaviour remains based on prior functional work. The study will be of interest to neuroscientists studying sensory processing and motor control, and provides an invaluable resource for future functional investigations.

# Referee report: Joint Public Review

**DOI:** 10.7554/eLife.108044.3.sa1

Summary:

Calle-Schuler et. al. reconstruct all the pre- and post-synaptic neurons to the bristle mechanosensory neurons on the adult fly head to understand if neural circuits support the parallel mechanosensory pathways, which could be instrumental in shaping the sequential motor patterns during fly grooming. They find that most presynaptic neurons, interneurons and excitatory post synaptic neurons are also somatotopically organized, such that each neuron is more connected to bristles mechanosensory neurons that are closer on the head and less connected to bristles mechanosensory neurons that are further away. These include the direct BMN-BMN circuits, excitatory interneurons, as well as the inhibitory networks. They also identify that the one entire hemi-lineage 23b form excitatory postsynaptic circuit with BMNs, highlighting how these circuits and hence their function could be developmentally determined.

Strengths:

This is a complete map of the all the neurons which make 5 or more pre- and post-synaptic connections of the fly head BMNs. Using this, the authors have identified various trends such as ascending neurons provide most of the GABAergic inhibitory input, which could provide the presynaptic inhibition essential for the parallel model for sequential grooming generation. Moreover, they identified that the entire cholinergic hemilineage 23b is postsynaptic to BMNs. Both their excitatory postsynaptic connectivity and inhibitory presynaptic connectivity demonstrate core motifs of the parallel circuits necessary for the hierarchical suppression model of grooming sequence.

Weaknesses:

Somatotropic organization with hierarchical suppression is an elegant mechanism to generate sequential motor sequence during grooming. Yet, anatomical connectivity alone, in absence of functional connectivity, cannot explain the grooming motor sequences. Future work should be aimed at mapping the functional connectivity with behavioral sequence.

Closing statement:

The authors have addressed the major concerns regarding clarity, scope, and interpretation. The manuscript is now significantly improved and is clearly framed as an anatomical resource that identifies circuit motifs consistent with existing models of grooming control.

# Author comment: Author response

**DOI:** 10.7554/eLife.108044.3.sa2

**Authors:** Steven A Calle-Schuler, Alexis Santana-Cruz, Lucia Kmecova, Stefanie Hampel, Andrew M Seeds

**Affiliations:** Institute of Neurobiology, University of Puerto Rico Medical Sciences Campus San Juan United States; Institute of Neurobiology, Department of Anatomy and Neurobiology, University of Puerto Rico Medical Sciences Campus San Juan United States; Institute of Neurobiology, University of Puerto Rico Medical Sciences Campus San Juan United States; Institute of Neurobiology, University of Puerto Rico Medical Sciences Campus San Juan United States; Institute of Neurobiology, University of Puerto Rico Medical Sciences Campus San Juan United States

The following is the authors’ response to the original reviews.

We sincerely thank the Reviewers for their careful reading and insightful critiques, which have helped make the manuscript clearer and more impactful.

In response to the Reviewers, we substantially revised the manuscript to improve clarity, framing, and accessibility for readers outside the *Drosophila* connectomics community, while keeping the core conclusions unchanged. We clarified the study’s scope (defining parallel circuit architecture rather than testing sufficiency for reconstructing grooming sequence order), restructured the last Introduction paragraph, several Results sections, and the Discussion to foreground the main findings and their relevance to the parallel hierarchical-suppression model. We also added key methodological clarifications for non-specialist readers, including how BMN classes were identified in FAFB by a correlative approach (with type-level, not single-bristle, resolution), how FlyWire/Codex synapse counts are defined (contacts vs T-bars), how sensory BMNs can have postsynaptic sites, and what is meant by ascending vs descending neurons in a brain-only dataset. Across the Results, we improved terminology and definitions (e.g., projection zones, hemilineage 23b, BMN nomenclature such as BM-InOm), clarified what derives from prior work (Eichler et al., 2024) versus new analyses, strengthened interpretation of BMN→motor connections as likely modulatory, and expanded explanation of postsynaptic partner categories. We also revised figures and legends to better highlight overlap/segregation and somatotopy, moved the cosine-similarity matrices into the main figures (new Figure 9), added a new graphical summary figure (new Figure 15), and explicitly acknowledged key limitations, including one-hemisphere analysis and lack of VNC coverage in FAFB.

In addition, in response to the suggestion of a rank-order test relating BMN→second-order wiring to the grooming hierarchy, we clarified throughout the revised manuscript that this study does not aim to test whether connectivity alone is sufficient to reconstruct grooming sequence order, and we removed wording that could imply such a claim. As detailed in our response to that specific critique below, sequence sufficiency is outside the scope of this study, and a simple linear ordering based on aggregate synapse weights is not straightforward to interpret in this system (e.g., BM-Taste vs. BM-InOm output strength does not track grooming order, BMNs likely contribute to multiple behaviors, and head grooming order is not resolved at sufficient granularity). We therefore respectfully request that the sentence in the eLife Assessment suggesting that the paper is weakened by not including this analysis be removed. As currently written, it frames an out-of-scope analysis as a missing test of the manuscript’s main claims and may mislead readers about the paper’s intended contribution: a synaptic-resolution anatomical definition of parallel BMN circuit architecture and motifs consistent with hierarchical suppression.

**Public Reviews:**

**Reviewer #1 (Public review):**

Summary:

Calle-Schuler et. al. reconstruct all the pre- and post-synaptic neurons to the bristle mechanosensory neurons on the adult fly head to understand how neural circuits determine the sequential motor patterns during fly grooming. They find that most presynaptic neurons, interneurons, and excitatory postsynaptic neurons are also somatotopically organized, such that each neuron is more connected to bristles mechanosensory neurons that are closer on the head and less connected to bristles mechanosensory neurons that are further away. These include the direct BMN-BMN circuits, excitatory interneurons, as well as the inhibitory networks. They also identify that the entire hemi-lineage 23b forms excitatory postsynaptic circuits with BMNs, highlighting how these circuits and hence their function could be developmentally determined.

Strengths:

This is a complete map of all the neurons that make 5 or more pre- and post-synaptic connections of the fly head BMNs. Using this, the authors have identified various trends, such as ascending neurons providing most of the GABAergic inhibitory input, which could provide the presynaptic inhibition essential for the parallel model for sequential grooming generation. Moreover, they identified that the entire cholinergic hemilineage 23b is postsynaptic to BMNs.

Weaknesses:

Although the somatotropic organization is an elegant mechanism to generate sequential motor sequences during grooming, none of the analyses in the paper directly demonstrate that this somatotropic connectivity is sufficient to generate hierarchical suppression and reconstruct the grooming sequence. If somatotropic organization is sufficient, then hierarchical clustering should recover the grooming sequence. Their detailed connectome enables the authors to test if some networks are more crucial for grooming sequence than others: to what extent can each network individually (ascending neurons-BMN alone) or a combination (BMN-BMN, ascending-BMN, BMN-descending, etc.) recover the sequence observed during grooming. If all the pre- and post-synaptic neurons put together cannot explain the sequence, then the sequence is probably determined by individual synaptic strengths or other key downstream neurons.

We appreciate the Reviewer’s interest in how BMN connectivity relates to the grooming sequence, and agree that understanding how mechanosensory circuits contribute to hierarchical action selection is an important direction. In this study, however, our goal was not to test whether connectivity alone is sufficient to reconstruct the full grooming sequence. Rather, we focused on defining the parallel circuit architecture underlying individual grooming movements and on identifying anatomical features—most notably extensive presynaptic inhibition—that are consistent with previously proposed models of hierarchical suppression.

We recognize that aspects of the Introduction and the references cited there to prior work on the grooming sequence may have led some readers to expect a direct sequence-prediction analysis. To address this, we revised the Introduction and Results to clarify the scope of the study and adjusted language to avoid implying that we aimed to derive the grooming order from connectivity. Consistent with this framing, the Abstract mentions the sequence only in the context of presynaptic inhibition, which provides anatomical support for existing models of hierarchical suppression. We therefore do not draw conclusions about the ordering of grooming movements from the connectome itself. Details of the specific manuscript revisions are provided below in the Recommendations for authors section.

The Reviewer suggests testing whether somatotopic organization is sufficient to recover the grooming sequence by clustering BMN connectivity or by examining whether specific subnetworks (e.g., BMN → ascending, BMN → descending, or BMN→BMN pathways) reproduce the sequence. We carefully considered these possibilities. However, several factors currently limit the interpretability of such analyses.

First, synaptic weight alone does not align with known features of the grooming sequence. For example, BM-Taste neurons contribute the majority of BMN synaptic output, yet proboscis grooming is not the first head grooming movement, whereas BM-InOm neurons contribute less than 9% of total output despite eye grooming occurring first. As we now clarify in the Results, global synapse number therefore does not predict the order of grooming movements.

Second, BMNs likely distribute signals across multiple behavioral pathways beyond grooming, including circuits involved in feeding and escape behaviors. Because the connectome aggregates all postsynaptic targets, analyses based solely on connectivity strength cannot isolate the subset of circuits specifically responsible for grooming-related action selection.

Third, the head grooming sequence itself has not been resolved at the spatial granularity required for such analyses across head regions. While eye grooming is well characterized as the first head movement, the relative ordering among antennae, proboscis, and other head bristle regions remains less clearly defined, making it difficult to evaluate correspondence between connectivity-derived rankings and behavioral order.

Because of these limitations, we concluded that clustering or network-based analyses aimed at reconstructing the grooming sequence from connectivity alone would be difficult to interpret and therefore chose not to include them. Accordingly, we have deliberately avoided claiming that the connectome is sufficient to generate the grooming sequence. Instead, we interpret the somatotopic architecture and inhibitory circuitry described here as anatomical features consistent with previously proposed models of hierarchical suppression, while leaving the question of sufficiency for future studies that integrate connectomics with functional and behavioral analyses.

Given that we do not claim sufficiency of the connectome for producing the grooming sequence, we respectfully request that the eLife Assessment avoid framing the manuscript around this expectation, as wording that implies the manuscript should reconstruct the sequence from connectivity could misrepresent the intended scope of the study and potentially mislead readers about its primary contributions.

**Reviewer #2 (Public review):**

Summary:

Schuler et al. present an extensive analysis of the synaptic connectivity of mechanosensory head bristles in the brain of *Drosophila melanogaster*. Based on the previously described set of bristle afferent neurons, (BMNs), located on the head, the study aims to provide a complete, quantitative assessment of all synaptic partners in the ventral brain. Activation of head bristles induces grooming behavior, which is hierarchically organized, and hypothesized to be grounded in a parallel cellular architecture in the central brain. The authors found evidence that, at the synaptic level, neurons downstream of the BMN afferents, namely the postsynaptic LB23 interneurons and recurrent GABAergic neurons (involved in sensory gain control), are organized in parallel, following the somatotopic organization described for the BMN afferents. This study, therefore, represents an important step towards a better understanding of the cellular circuits that govern the hierarchical order of sequentially organized grooming behavior in *Drosophila melanogaster*.

The study is well done, the images are well designed and extensive in number, but the account is challenging to read and digest for the reader outside the Drosophila /connectome community. It is amazing what can be done with the connectome nowadays using the up-to-date FAFB dataset, the analytical and visual tools (as in FlyWire), in combination with known anatomy/physiology/behavior in DM. I suggest that the authors provide more detail on hemilineages, their relationship to the FAB connectome, the predicted neurotransmitter identity, and the use of statistical CatMAID tools used in some of the Figures.

A graphical summary at the end of the study would be very useful to highlight the important findings focusing on neuron populations identified in this study and their position in the hypothesized parallel central circuitry of BMNs.

We thank the Reviewer for the thoughtful and constructive comments. In response, we substantially revised the manuscript to improve clarity and accessibility, particularly for readers outside the *Drosophila* connectomics community. We rewrote portions of the Introduction, Results, and Discussion to better foreground the main findings, reduce density, and more clearly distinguish prior work from the new analyses presented here. We also added methodological clarification throughout, including how BMN classes were identified in the FAFB dataset using a correlative, type-level approach, how FlyWire/Codex synapse counts are defined, and clarified terminology related to projection zones, pre- versus postsynaptic structure, and partner classes. To address the Reviewer’s request for more developmental context, we added a more explicit definition of hemilineages at first mention in the Abstract and Results. In addition, we revised figures and legends to make the somatotopic and parallel organization of the circuitry easier to interpret, including moving the cosine-similarity matrices into the main figures. Finally, in direct response to the Reviewer’s suggestion for a higher-level synthesis, we added a new graphical summary figure (Figure 15) at the end of the manuscript to highlight the principal neuron populations identified in the study and their proposed positions within the parallel central BMN circuitry. Together, we believe these revisions have made the manuscript clearer, more accessible, and better framed for a broad readership while preserving its core conclusions. Details of these changes are provided in the Recommendations for the authors section.

**Reviewer #3 (Public review):**

Summary:

The authors set out to extend their previous mapping of Drosophila head mechanosensory neurons (Eichler et al., 2024) by reconstructing their full second-order connectome. Their aim is to reveal how bristle mechanosensory neurons (BMNs) interface with excitatory and inhibitory partners to generate location-specific grooming movements, and to identify the circuit motifs and developmental lineages that support this transformation.

Strengths:

The strengths of this work are clear. The authors present a comprehensive synaptic-resolution connectome for BMNs, identifying nearly all of their pre- and postsynaptic partners. This dataset reveals important circuit motifs:

(1) BMNs provide feedforward excitation to descending neurons, feedforward inhibition to interneurons, and are themselves strongly regulated by GABAergic presynaptic inhibition.

(2) These motifs together support the idea that BMN activity is locally gated and hierarchically suppressed, fitting well with known behavioural sequences of grooming.

(3) The study also shows that connectivity preserves somatotopy, such that BMNs from neighbouring bristle populations converge onto shared partners, while distant BMNs remain segregated.

(4) A developmental analysis reveals both primary and secondary partners, suggesting a layered scaffold plus adult-specific elaborations.

(5) Finally, the identification of hemilineage 23b (LB23) as a core postsynaptic pathway - incorporating previously described antennal grooming neurons (aBN2) - provides a striking link between developmental lineage, anatomical connectivity, and behavioral output.

(6) Together, the dataset represents a valuable resource for the neuroscience community and a foundation for future functional studies.

Weaknesses:

There are also some weaknesses that mostly only limit clarity.

(1) The writing is dense, with results often presented in a cryptic fashion and the functional implications deferred to the discussion. As a result, the significance of circuit motifs such as BMN→motor or reciprocal inhibitory loops is sometimes buried, rather than highlighted when first described.

We thank the Reviewer for this helpful suggestion. In response, we revised several sections of the Results to improve clarity and more clearly highlight the functional significance of key circuit motifs when they are first introduced. Specifically, we streamlined dense passages and added brief explanatory statements linking motifs such as reciprocal inhibitory loops to their potential roles in the proposed parallel circuit architecture. Additional details of these revisions are provided in the Recommendations for the authors section below.

(2) Some assumptions require more explanation for non-specialist readers - for example, how bristle identity is inferred in EM in the absence of cuticular structures, or what is meant by "ascending" and "descending" in a dataset that does not include the ventral nerve cord. While some of this comes from the earlier paper, it would help readers of this one to explain this.

In response, we added clarifying text describing how BMN types were identified in the FAFB dataset using a correlative approach based on stereotyped projection morphologies and prior light-level anatomical data, and we explicitly state the limits of this type-level assignment in the absence of cuticular bristles in the EM volume. We also expanded the explanation of partner categories, including what is meant by “ascending” and “descending” neurons in a brain-only dataset. Additional details of these revisions are provided in the Recommendations for the authors section.

(3) Visualization choices also sometimes obscure key conclusions: network graphs can be visually appealing but do not clearly convey somatotopy or BMN-type differences; heatmaps or region-level matrices would make the parallel, block-like organization of the circuit more evident.

We incorporated connectivity matrices (cosine-similarity heatmaps) into the main figures to more clearly illustrate the somatotopic and parallel organization of BMN connectivity, complementing the network graph visualizations (new Figure 9). These matrices make the block-like structure of BMN partner relationships more apparent and help highlight differences among BMN types; additional details are provided in the Recommendations for the authors section.

(4) The data might also speak to roles beyond grooming (e.g., mechanosensory modulation of posture or feeding), and a brief acknowledgement of this would broaden the impact.

We added text acknowledging that BMNs contribute to additional behaviors beyond grooming, such as feeding and other mechanosensory-guided actions. These roles are supported by prior studies of bristle function and are also consistent with the diverse downstream circuits revealed in the connectome. This clarification broadens the interpretation of the dataset while maintaining the primary focus of the study on grooming-related circuitry.

(5) The restriction to one hemisphere should be explicitly acknowledged as a limitation when framing this as a 'comprehensive' connectome.

We thank the Reviewer for this suggestion. We now explicitly acknowledge this limitation in both the Results and Discussion.

In the Results section entitled “The BMN connectome” we added a sentence at the end of the paragraph that mentions the limitations. This sentence reads: “In addition, because our analysis was restricted to BMNs entering the left hemisphere, the complete right-side BMN connectome is not included, limiting assessment of bilateral symmetry, inter-hemispheric coordination, and variability across sides.”

The last paragraph of the first Discussion section describes limitations to our ‘comprehensive’ connectome. The text in this paragraph pertaining to the left/right variability reads: Second, the analysis focuses only on BMNs from the left hemisphere. Although contralateral neurons synapsing with left-side BMNs are included, the absence of the right-side BMN connectome limits assessment of bilateral symmetry, interhemispheric coordination, and side-to-side variability.

Overall, the authors achieve their main goal: they convincingly show that BMNs connect into parallel, somatotopically organized pathways, with LB23 providing a key lineage-based link from sensory input to grooming output. The dataset is carefully analyzed, and while the presentation could be streamlined, the connectome will be a valuable resource for researchers studying sensory processing, motor control, and the logic of circuit organization.

**Recommendations for the authors:**

**Reviewing Editor Comments:**

We enjoyed this work and are enthusiastic about its contribution: the resource is valuable, and the anatomical evidence is solid. Most of our suggestions concern clarity and visualization, detailed below.

In addition, the editors and reviewers felt one focused analysis would materially strengthen the paper: please use the BMN→second-order synapse weights to produce a similarity-based, one-dimensional order of BMN types and test its agreement with the known grooming sequence (e.g., via a rank correlation). A positive result would support sufficiency of the mapped wiring for the sequence; if not, the claims can be framed as "consistent with" rather than "sufficient for."

We appreciate the Reviewers’ interest in how BMN connectivity relates to the grooming sequence, and agree that understanding how mechanosensory circuits contribute to hierarchical action selection is an important direction. In this study, however, our goal was not to test whether connectivity alone is sufficient to reconstruct the full grooming sequence. Rather, we focused on defining the parallel circuit architecture underlying individual grooming movements and on identifying anatomical features—most notably extensive presynaptic inhibition—that are consistent with previously proposed models of hierarchical suppression.

We recognize that references in the Introduction to prior work on the grooming sequence may have led some readers to expect a direct sequence-prediction analysis. To address this, we revised the Introduction and Results to clarify scope and adjusted language to avoid implying that we aimed to derive the grooming order from connectivity. Consistent with this framing, the Abstract mentions the sequence only in the context of presynaptic inhibition, which provides anatomical support for existing models of hierarchical suppression. We do not draw conclusions about the ordering of grooming movements from the connectome itself.

The Reviewer-suggested analysis—using BMN-to-partner synaptic weights to derive a linear ordering of BMN types—is conceptually reasonable, but its interpretability is limited at present. First, synaptic weight alone does not align with known features of the grooming sequence: BM-Taste neurons contribute the majority of BMN synaptic output, yet proboscis grooming is not the first head movement, whereas BM-InOm neurons contribute less than 9% of output despite eye grooming occurring first. Second, BMNs likely project to multiple pathways supporting distinct behaviors, such as feeding and escape, complicating any attempt to infer a single grooming hierarchy from aggregate connectivity. Third, the head grooming sequence itself has not been resolved at the granularity required for such an analysis, particularly among the antennae, proboscis, and other head bristle regions. Accordingly, we have deliberately refrained from making claims that connectivity is sufficient to generate the grooming order.

Given that we do not claim sufficiency of the connectome for producing the grooming sequence, we respectfully request that this point be removed from the public eLife Assessment, as its current wording implies an unmet expectation outside the intended scope of the study and could mislead readers about the manuscript’s primary contributions. We appreciate the opportunity to clarify our framing and to ensure that the goals and outcomes of the work are accurately represented.

Revisions.

(1) Gave the last paragraph of the Introduction more structure to clearly state the main findings of the study in the context of what we learned about the circuit architecture proposed by the parallel model of hierarchical suppression.

New paragraph: “Here, we define the synaptic connectivity of head BMNs by mapping nearly all of their pre- and postsynaptic partners—including other BMNs, ascending and descending neurons, interneurons, and motor neurons—within the FAFB dataset. Consistent with a parallel model, we find that both presynaptic and postsynaptic partners are somatotopically organized, preserving the spatial layout of the bristle map and revealing a set of parallel mechanosensory pathways that correspond to distinct head regions. Within the postsynaptic population, we identify the developmentally-related cholinergic hemilineage 23b (LB23), whose members exhibit region-specific BMN connectivity and include neurons previously shown to elicit aimed head grooming movements when activated. This demonstrates how LB23 neurons participate in parallel postsynaptic pathways that may drive discrete components of head grooming. On the input side, BMNs receive substantial presynaptic inhibition from predominantly GABAergic partners, providing strong feedback and feedforward control over mechanosensory signaling. This inhibitory architecture is consistent with hierarchical-suppression models in which inhibition regulates sensory gain and prioritizes competing actions in the grooming sequence. Together, this mechanosensory connectome reveals core organizational principles—parallel somatotopic architecture, region-specific excitatory pathways, and strong inhibitory regulation—that are thought to constitute foundational circuit motifs supporting head grooming.”

(2) In the Results section entitled “BMN synapses show large quantitative variation across types”, we added text to the third paragraph that makes it clear that raw synapse numbers alone do not predict the sequence, if one just compares the first movement (eye grooming) and a later movement in the sequence (proboscis grooming).

That text reads: “Notably, if grooming order were driven simply by relative sensory drive—i.e., by BMN types with the strongest synaptic output eliciting cleaning of their corresponding locations first—then synapse number should track the grooming sequence. Instead, differences in synapse number do not align with the order of the grooming sequence: BM-Taste neurons account for the majority of BMN output, yet proboscis grooming is not the first head grooming movement performed, whereas BM-InOm neurons contribute only a small fraction of output despite eye grooming occurring first (Figure 1E, Figure 2A,B). This indicates that global synapse number alone is not a reliable predictor of the grooming sequence.”

(3) In the results section entitled “BMN postsynaptic partners are excitatory and inhibitory”, we added text to two different sentences to better link the results with what we are trying to test with respect to the parallel model of hierarchical suppression.

Modified sentence 1: “This excitation is hypothesized in the parallel model to help form BMN feedforward circuits that elicit aimed grooming of specific body locations, while feedforward inhibition could mediate suppression of competing grooming movements (Figure 1 – figure supplement 1A, B).”

Modified sentence 2: “Taken together, the BMN postsynaptic partners include a diverse set of neurons that mediate both feedforward excitation and inhibition and feedback inhibition, features predicted by the parallel model.”

(4) In the Results section entitled “BMNs and LB23 neurons form somatotopic pathways that elicit aimed grooming, we added text to the first sentence that better ties the section to the overall goals of the manuscript.

That text now reads: “In accordance with the parallel model of grooming, we hypothesize that BMNs connect with somatotopically organized excitatory parallel pathways eliciting aimed grooming of specific head locations (Figure 1 – figure supplement 1A, C).”

**Reviewer #1 (Recommendations for the authors):**

(1) The connectivity matrix (like that in Lesser et al., 2024, Nature, and also in Figure 9, Figure Supplement 1 of this paper) is an easier-to-digest representation of the various connections shown in Figure 2.

We agree that connectivity matrices provide a clearer and more accessible representation of these data. Based on the context of this and other comments, we understand the Reviewer to be referring to Figure 9 rather than Figure 2. In response, we have moved the cosine-similarity connectivity matrices previously shown in Figure 9 – figure supplement 1 into the main manuscript, where they now appear as Figure 9.

These matrices depict similarity among BMN postsynaptic partners. At present, we are unable to generate equivalent matrices for presynaptic partners due to recent personnel constraints in the lab. For this reason, we have retained the original network-graph representation (now Figure 10) to display the full pre- and postsynaptic connectome structure.

We hope this compromise addresses the Reviewer’s request while clearly presenting the available analyses.

(2) Again, "Cosine based clustering is essential to demonstrate the somatotropic organization" the data in Figure 9 - Figure Supplement 1 demonstrates this better than the main Figure 9. This supplementary figure would be a great addition to the main manuscript.

Please see the preceding response for details on the changes that we made to address this reviewer comment.

(3) Figure 9 - Figure Supplement 1A: Can the authors explain why the InOm occur in two clusters (red in top and bottom)? Do InOm neurons show two different kinds of connectivity patterns?

This is a great question! We had written a possible explanation for this in the Discussion section entitled “A synaptic resolution connectome of a head somatotopic map”.

“One notable exception to this pattern is the BM-InOm population, which occupies a central position in network diagrams and exhibits broad connectivity similarity with BMNs from across the head (Figure 9A, Figure 10A-E). This likely reflects the large surface area of the compound eyes, which span dorsal, ventral, and posterior regions and neighbor multiple bristle populations. Consistent with previous work showing morphological diversity among BM-InOm neurons (Eichler et al., 2024), our output connectivity analysis suggests the presence of multiple BM-InOm subtypes defined by distinct partner profiles (Figure 9A). Future work will be needed to determine how this heterogeneity relates to spatial organization within the eye.”

**Reviewer #2 (Recommendations for the authors):**

All further comments for the authors are aimed at a better understanding of the text and for clarity. The manuscript needs revision.

(1) Ventral brain:

Please specify this term. Is it the SEG, or the gnathal ganglion? Throughout the paper, 'ventral brain', or 'brain', is the only anatomical terms you use. Are all pre-/post- partners of BMNs located in this region? I understand that you provide a statistical analysis on a network level, here, but as far as I know, the neuropil regions in Drosophila are reported in more detail on the macroscopic level (see, e.g., Itoh).

Based on our understanding of the Ito et al reference, SEG was “retired” in that manuscript in favor of gnathal ganglia. We considered using the term subesophageal zone (SEZ) in the manuscript, but ultimately chose not to adopt it. In the *Drosophila* brain nomenclature (Ito et al., 2014), the SEZ is defined as a region below the esophagus that encompasses multiple neuropils, such as the gnathal ganglia (GNG) and saddle (SAD), rather than a single anatomically discrete structure.

In our dataset, the GNG are the ventral-most neuropil containing the BMN projections and the highest density of BMN-related synapses, and we therefore refer to this structure explicitly where appropriate. However, BMN pre- and postsynaptic partners are not confined to the GNG or to the SEZ as a whole; some partner neurites extend dorsally into additional neuropils. As a result, the term SEZ does not accurately capture the full spatial extent of the BMN connectome analyzed here.

For clarity and consistency across analyses that span multiple adjacent neuropils, we therefore use the broader functional descriptor “ventral brain”, while explicitly identifying the gnathal ganglia and other neuropils when discussing neuropil-level synapse distributions. We believe this approach most accurately reflects both the anatomical organization of the circuit and the scope of our analysis.

Given this Reviewer’s comment, we anticipate that not mentioning the SEZ in this manuscript might result in similar confusion among readers of our manuscript. Therefore, we now mention the SEZ and the supraesophageal zone (SPZ) at the end of the Results section entitled “Synapses of BMN partners are mostly concentrated in the ventral brain”. We also added the SEZ and the SPZ to the new last summary figure (Figure 15) to help clarify the locations of the BMNs and their second order connectome.

That text reads: “Thus, while most neuropils containing synapses of second-order BMN partners are located below the esophagus (in the subesophageal zone, SEZ), we found more limited involvement of neuropils in the supraesophageal zone (SPZ; above the esophagus), suggesting relatively limited direct top-down control.”

(2) Please provide greater clarity in your use of the terms synapse-presynapse-pre- and postsynaptic partners:

In insects, synapses are polyads. It is therefore essential to distinguish whether by presynaptic (pre) you mean 1. the number of T-bars (presynaptic sites) or 2. the number of (outgoing) synaptic contacts made by a single presynaptic T-bar site. For example, a synapse configured as a tetrad (a polyad) consists of one presynaptic T-bar opposed to four postsynaptic profiles and can be counted either as one synapse (one presynaptic site, one T-bar, in CATMAID: a presynaptic connector) OR as four (outgoing) synaptic connections since the single T-bar connects to four different postsynaptic profiles. This distinction is crucial for quantifying synaptic networks in insects. Thus, the "number of synapses" may refer to 1. The number of presynaptic sites = number of T-bars = number of polyads formed by a particular neuron. 2. the number of actually outgoing synaptic contacts, a number that also reflects the degree of polyadicity. 3. number of postsynaptic sites (that is easy).

This distinction (regarding the counts of presynapses) was reported in previous connectome studies (e.g., Horne, 2018; Gruber, 2025; Schlegel,2023). Schlegel notes: ' Insect synapses are polyadic, i.e., each presynaptic site can be associated with multiple postsynaptic sites. In contrast to the Janelia hemibrain dataset, the synapse predictions used in FlyWire do not have a concept of a unitary presynaptic site associated with a T-bar. Therefore, presynapse counts used in this paper do not represent the number of presynaptic sites but rather the number of outgoing connections.' End of citation from Schlegel.

We thank the Reviewer for highlighting this important distinction. We now clarify in the Materials and methods that synapse counts are based on Codex/FlyWire annotations, which report individual pre- and postsynaptic contacts rather than unitary presynaptic sites (T-bars), consistent with prior FlyWire-based connectome studies (e.g., Schlegel et al.). We also added a brief clarification in the Results indicating that pre- and postsynaptic numbers refer to incoming and outgoing contacts.

We added a sentence to the first section of the Materials and methods entitled “Connectome data and neuron meshes”. This text reads: “Synapse counts throughout this study are based on FlyWire/Codex synapse annotations and represent the number of individual pre- to postsynaptic contacts (incoming or outgoing connections), rather than the number of presynaptic active sites (T-bars); thus, presynaptic counts reflect polyadic connectivity as described previously (Schlegel et al., 2023).”

(3) In your study, a potential misunderstanding of this distinction arises when comparing statements on line 168 versus line 184:

On line 168, you state: '... each BMN type having .... more postsynaptic than presynaptic sites'. However, on line 184 you state: 'There were significantly more postsynaptic than presynaptic partners, in agreement with the BMNs containing more presynaptic than postsynaptic structures. These are contradictory: the statement on line 168 seems to refer to the number of presynaptic T-bars, while on line 184 you refer to the number of actually outgoing connections (which more accurately reflects the degree of polyadicity). Since BMNs are sensory afferent, they are indeed expected to have more outgoing synapses into the central brain.

We thank the Reviewer for identifying this mistake. We have revised the sentence at former line 168 to now read: “In addition to differing in total synapse number, BMN types vary in their pre- versus postsynaptic composition: all BMNs contain both (Eichler et al., 2024), with presynaptic sites outnumbering postsynaptic sites by ~2× to ~9× across types (mean ≈5:1 output-to-input ratio, Figure 2 – figure supplement 1A, B, Supplementary file 2, Supplementary file 3).”

(4) Identification of bristle sensory afferents in the brain:

This is explained in more detail in the Eichler paper, but not here. I do not understand how you identified these neurons in the FAFB dataset. The number and distribution of the individuum of the FABF EM dataset are not known, and because there is variability in the number of bristles in individual flies, the true number of bristle neurons for synaptic analysis can only be estimated. The correlative approach necessary to find the bristle sensory neurons in the FAFB set is still unclear to me. See also my comments on Figure 1.

We thank the Reviewer for raising this point. We agree that our original draft did not clearly explain the correlative approach used to identify head BMNs in the FAFB dataset, and we have revised the manuscript to make this workflow explicit.

In our prior work (Eichler et al., 2024), we quantified the number of bristles in each head bristle population and assessed the extent to which populations are invariant versus variable across individuals. This established an expected range for BMN counts by bristle population and clarified the level of variability that can be expected biologically.

We then identified BMN types corresponding to specific bristle populations using different techniques, such as dye fills and light microscopy, which allowed us to define the characteristic projection morphologies and CNS entry routes associated with each population. These light-level anatomical signatures provided the basis for locating the corresponding axons in the FAFB EM volume and reconstructing the same neuron classes in EM. Importantly, because bristles themselves are not present in the EM volume, this approach supports type-level assignment (bristle population/BMN class) rather than single-bristle resolution, and we now state this explicitly to avoid overinterpretation.

To ensure this is clear to readers who have not read Eichler et al., we have added explanatory text in the Results and expanded the Figure 1 legend describing: (i) how BMN types were identified and matched, (ii) what can and cannot be resolved given natural bristle-number variability, and (iii) how this impacts interpretation of “completeness” at the level of BMN types rather than individual bristles.

In paragraph 1 of the first Results section, entitled “BMN synapses are somatotopically distributed in the ventral brain”, we added text that briefly describes the previous linkage of the head BMNs to the FAFB dataset. That text reads: “In prior work (Eichler et al., 2024), we showed that head bristle populations are innervated by specific BMN types whose axons project to distinct, spatially localized regions (projection zones) in the ventral brain (Figure 1C,D, left, Figure 1 – figure supplement 2A-E). This was determined using dye fills and light-microscopy-based tracing to identify BMN types innervating defined head bristle populations and to establish their characteristic brain projection morphologies. Bristle population counts and their variability across individuals provided expectations for BMN number per type. This quantitative constraint, combined with the highly stereotyped projection morphologies, provided a correlative anatomical framework to locate and reconstruct nearly all BMNs in the FAFB serial-section EM volume and map their projections into the CNS. Because FAFB does not include the head cuticular bristles, individual BMNs could not be linked to single bristles. Therefore, these assignments are necessarily correlative and provide type-level (population) rather than single-bristle resolution. Nevertheless, this level of resolution was sufficient to define somatotopically organized projection zones."

(5) Results:

(a) Line 102: explain hemilineage 23 B

We added text in the manuscript to better define hemilineages.

In the Abstract, we added to a sentence that highlights that the LB23 neurons are developmentally related. That sentence now reads: “We identified an excitatory cholinergic hemilineage (hemilineage 23b), a developmentally related group of neurons that elicits aimed head grooming and exhibit differential connectivity with BMNs from distinct head locations, revealing a lineage-based somatotopically organized parallel circuit architecture.”

Results section entitled “The entire cholinergic hemilineage 23b (LB23) is postsynaptic to BMNs”, we added a sentence that defines hemilineage at its first mention in the Results section. We also made slight modifications to the preceding and following sentences. That text reads: “To identify neurons crucial for establishing the BMN-postsynaptic parallel pathways that elicit head grooming movements, we focused on secondary hemilineages. In the *Drosophila* CNS, a hemilineage refers to the cohort of neurons derived from a single stem cell-like neuroblast that share a common developmental origin, stereotyped morphology, and are thought to have related functional roles within a circuit (Harris et al., 2015; Wreden et al., 2017). This focus was motivated by earlier findings that neurons whose activation elicited head grooming had morphologies consistent with specific hemilineages (Hampel et al., 2015; Seeds et al., 2014).”

(b) Line 151: - line 171: it is not clear to me what a projection zone is.

We thank the Reviewer for raising this point. We agree that the term “projection zone” benefits from a brief clarification. We have made minor edits at two locations to explicitly state that projection zones refer to spatially localized regions of BMN axonal arborization and synaptic distribution corresponding to specific head locations.

Changes made in the manuscript:

A sentence that first introduces the term in the fourth paragraph of the Introduction now reads: “Indeed, the BMN axon projections in the central nervous system (CNS) show a somatotopic arrangement, where distinct projection zones—spatially localized regions of axonal arborization and synaptic output—correspond to specific head and body locations (Eichler et al., 2024; Johnson and Murphey, 1985; Murphey et al., 1989; Newland, 1991; Newland et al., 2000; Tsubouchi et al., 2017).”

In a sentence in the first paragraph of the first Results section, we added a brief clarifying definition of “projection zones” at their first mention in the Results. That sentence reads: In prior work (Eichler et al., 2024), we showed that head bristle populations are innervated by specific BMN types whose axons project to distinct, spatially localized regions (projection zones) in the ventral brain (Figure 1C,D, left, Figure 1 – figure supplement 2A-E).

(c) Input-output versus presynapse-postsynapse?

A revised sentence in the last sentence of the Results section makes this distinction clear: In addition to differing in total synapse number, BMN types vary in their pre- versus postsynaptic composition: all BMNs contain both (Eichler et al., 2024), with presynaptic sites outnumbering postsynaptic sites by ~2× to ~9× across types (mean ≈5:1 output-to-input ratio, Figure 2 – figure supplement 1A,B, Supplementary file 2, Supplementary file 3).

(6) Figures:

For clarity, it would be helpful if you indicated by the arrow the name of the sensory location (antenna, eye, etc.).

We appreciate this suggestion. Major sensory locations corresponding to different head bristle populations are indicated in Figure 1 – figure supplement 1C. We explored adding these labels directly to Figure 1A, but found that doing so made the panel overly crowded and less clear. To improve visibility while keeping the main figure uncluttered, we now explicitly direct readers to this figure supplement in the Introduction.

Specifically, we added a reference to Figure 1 – figure supplement 1C in the following sentence in the Introduction: Dust-induced head grooming is performed by the forelegs that start with the eyes and progress to other locations such as the proboscis and antennae (major head locations shown in Figure 1 – figure supplement 1C) (Seeds et al., 2014).

(a) Figure 1:

A: the presence of bristle types on the head. Are the JO afferents you mention in the text reported here?

Figure 1 does not include the JONs, which were described in detail in our previous study (Hampel et al., 2020).

The JONs are mentioned in the Figure 1 – figure supplement 1. We have added text to this legend to indicate that the JONs are not the subject of this study. This text reads: “(C) Mechanosensory neurons from different head locations project to distinct, somatotopically organized zones in the ventral brain and elicit aimed grooming of those locations, including the antennae (via JONs [Johnston’s organ neurons; not analyzed in this study] and BMNs), eyes (BMNs), and proboscis (BMNs).”

Are the reconstructions shown 1 B-D also from the Eichler paper?

We regret that this was not explicitly stated in the figure legend, and have revised the legend to distinguish between what was previously published and what is new to this study.

In the Figure 1 legend, we revised the following sentence: (C, D) Reconstructed BMN projections in the ventral brain (left, previously described in (Eichler et al., 2024)) and their corresponding pre- and postsynaptic sites (right, this study), colored by type according to the bristles that they innervate.

To make this clearer in the main text, we have rewritten the first sentence in the first paragraph of the Results: In prior work (Eichler et al., 2024), we showed that head bristle populations are innervated by specific BMN types whose axons project to distinct, spatially localized regions (projection zones) in the ventral brain (Figure 1C,D, left, Figure 1 – figure supplement 2A-E).

The dots are symbolic, or do they represent the number of bristles? The number of bristles cannot be identified, and thus stems from the FABF dataset.

The dots are symbolic and do not represent the number of bristles in the FAFB dataset. As noted in response to a related reviewer comment above, the numbers and variability of head bristles were quantified in our prior work (Eichler et al., 2024). We also used dye fills and light-microscopy approaches, which provided the framework for linking BMN types to bristle populations. We have clarified this point in the revised manuscript, as described in the response above.

Synapse number of bristle afferents: number of all pre-and postsynaptic contacts?

We have addressed this point above.

(b) Figure 2:

Again, the term synapses refers to all pre-and postsynaptic contacts ?

The Figure 2 legend indicates that synapse numbers include both input and output synapses. Additionally, now the first reference to Figure 2 indicates that numbers refer to both input and output synapses.

(c) Figure 2:

Supplement presynaptic/postsynaptic means pre- and post partner?

Presynaptic: number of BMNs that were connected with at least 5 synapses to any given presynaptic partner (n), the numbers of synaptic inputs to BMNs (inputs), and the number of presynaptic partners (partners). Postsynaptic: number of BMNs that were connected with at least 5 synapses to any given postsynaptic partner, the numbers of synaptic outputs to postsynaptic partners, and the number of postsynaptic partners.

(d) Figure 3:

Explain downstream-upstream

Downstream refers to postsynaptic while upstream refers to presynaptic partners or pathways.

Comparing the right side of the Sankey d. with your diagram in B, just by judging, I see more partners of descending (post) than interneurons (post) in A. However, in B, there are clearly more postsynaptic interneurons than descending posts? There are no numbers in Figure 3A.

This is a great point! Figure 3A (the Sankey diagram) summarizes the fraction of BMN synaptic output distributed across partner classes, normalized within each BMN type. In this representation, descending neurons occupy a larger fraction because, across BMN types, they collectively receive a higher proportion of BMN output synapses.

In contrast, Figure 3B (the sunburst plot) summarizes the number of distinct postsynaptic partner neurons in each category. Here, interneurons are more numerous than descending neurons, even though individual interneurons tend to receive fewer BMN synapses on average.

Thus, the two plots are consistent: descending neurons are fewer in number but receive more synapses per neuron, whereas interneurons are more numerous but receive fewer synapses per neuron on average. When postsynaptic synapse counts are summed (as in the bottom plots), the totals for descending neurons and interneurons can therefore appear similar, despite their different representations in the Sankey diagram.

We have added text in the Results section entitled “BMN synaptic partners in the CNS: ascending, descending, and interneurons”. Text was added here because it also nicely responds to another Reviewer comment below for more description of the postsynaptic partners. That added text reads: “Interneurons are more numerous as distinct partner neurons, whereas descending neurons receive a larger fraction of BMN output synapses across BMN types (Figure 3A,B). Thus, descending neurons are fewer in number but tend to receive more BMN synapses per neuron on average, while interneurons are more numerous but often receive fewer synapses per neuron.”

(e) Figure 10: I cannot see colored circles. I found Figure 10 very hard to understand. Is this a visualization created in CATMAID? As I mentioned before, a graphical summary highlighting the information flow and architecture of the circuits analyzed in this study would be useful. In such a diagram, you could combine the findings of your study, the open question, and the undeciphered pathways. In short, a schematic of the current knowledge of the potentially parallel and recurrent architecture of the BMN circuitry.

Figure 10 (now Figure 11) is intended to specifically examine neurons that are both pre- and postsynaptic to BMNs, rather than to summarize the full connectome. The goal of this figure is to highlight two features of pre/post neurons: their somatotopic connectivity with BMN types and the presence of bilaterally symmetric neuron pairs that connect to common BMN populations.

This visualization was generated from connectome-derived connectivity data and not from CATMAID, although it uses neuron reconstructions and synapse annotations from the FAFB dataset. The colored nodes represent BMN types and are now consistently referred to as “dots” rather than “circles” to better match their appearance. We have simplified the figure legend to clarify these points.

In response to this and related comments, we also added a new graphical summary figure (Figure 15) at the end of the manuscript that schematically summarizes the information flow and parallel, recurrent architecture of the BMN circuitry at a higher level.

(7) Discussion:

I found the first part of your discussion hard to read; the second part is better. You can condense the discussion by mentioning the results/hypothesis of previous work once, and avoiding repetitions, such as the uniqueness of the BMN connectome/FAB dataset.

In response to this comment, we condensed the opening portion of the Discussion by reducing repetition of background and prior findings, particularly references to earlier BMN work and the uniqueness of the FAFB dataset. We streamlined overlapping sections, mentioned prior hypotheses and results only once, and focused the revised text more directly on the new contributions of this study—namely, the synaptic-resolution organization, somatotopic connectivity, and circuit principles revealed by the BMN connectome.

There are several cases of vague sentences, e.g.: (a) Line 827: 'Head BMNs project from bristles to somatotopically organized zones in the brain (? ventral brain ?), with those innervating neighboring populations (? of bristles ?) occupying overlapping zones (Figure 1A-D)'.

We made this suggested change: Head BMNs project from bristles to somatotopically organized zones in the ventral brain, with those innervating neighboring bristle populations occupying overlapping zones (Figure 1A-D).

A remark: maybe you should indicate in Figure 1D the overlapping and segregated zones. The resolution is very low in these images.

We thank the Reviewer for this comment and agree that overlap versus segregation of projection zones was not sufficiently guided in the original presentation. Rather than adding arrows to Figure 1C,D, which we felt would reduce clarity, we now explicitly describe how overlap and segregation can be identified based on color mixing of BMN synapses in the text and figure legend. In addition, we highlight these features more clearly in Figure 1 – figure supplement 3, which provides higher-resolution, multi-view visualizations of BMN synapses where overlap and non-overlap are most evident.

Results:

Segregation between projection zones is apparent where synapses of distinct BMN types occupy non-overlapping regions with little or no color mixing, whereas overlap between projection zones is visible as spatial intermixing of differently colored synapses from neighboring BMN types (Figure 1C, D, right, Figure 1 – figure supplement 3A-E).

Figure 1 legend:

Overlapping projection zones are evident where synapses of different BMN types spatially intermingle, whereas segregated zones show little or no color mixing.

Figure 1 – figure supplement 3 legend:

These views highlight both overlapping projection zones, visible as intermingled synapses of different colors from neighboring BMN types, and segregated zones, where synapses from distinct BMN types remain spatially separated with minimal color mixing.

(b) Line 860: What is: 'location groomed'?

Added a clarification to this sentence: Thus, the location groomed (i.e. antennae) corresponds to the location of the majority of BMN inputs.

(c) Line 944: 'The sensory to motor resolution' What do you mean, here?

We have revised this sentence to “The spatial resolution of the sensory-to-motor transformation in this parallel circuit architecture remains to be tested.”

(d) The term: 'neighboring bristles' is unclear. Does it mean 'neighbor relates to members within he same bristle type (antennae)', or 'bristles of different types', e.g. antennae and eye bristles.

We thank the Reviewer for raising this point. Throughout the manuscript, the term “neighboring bristles” is used primarily to refer to neighboring bristle populations (i.e., bristles from different anatomical groups that are spatially adjacent on the head). In some contexts, the term is also used more generally to describe spatial proximity, regardless of whether the bristles belong to the same or different populations. Importantly, in both cases, the usage reflects the same underlying observation: BMNs innervating bristles that are spatially closer—whether within or between populations—show greater similarity in their postsynaptic connectivity than BMNs innervating more distant bristles.

(e) Avoid abbreviations, or explain shortly, the term under discuss: line 725: BMlnOm?

We thank the Reviewer for pointing out that the BMN nomenclature was not sufficiently clear. BMNs are named according to the bristle population they innervate (e.g., BM-Ant neurons innervate antennal bristles; BM-InOm neurons innervate interommatidial eye bristles), as defined in the Figure 1 legend. To improve clarity, we ensured that the first occurrences of these terms in the Results explicitly include the corresponding head location (e.g., “eye BM-InOm neurons”), and we added brief contextual reminders at later points where this abbreviation appears. These changes clarify the meaning of BM-InOm and related abbreviations without introducing additional terminology.

Changes made:

Figure 1 legend: clarified that BMNs are named according to the bristle population they innervate (e.g., BM-Taste neurons innervate Taste bristles).

Results, early first section (second paragraph): added head-location qualifiers at first mention (e.g., “eye BM-InOm neurons,” “proboscis BM-Taste neurons”) in sentences such as: “35 BM-Taste neurons innervating Taste bristles on the proboscis…” and “405 eye BM-InOm neurons innervating the interommatidial bristles on the eyes…”.

Later Results text where the abbreviation appears (including the sentence addressing the 5-synapse cutoff): added “eye” before BM-InOm for context (e.g., “although 555 eye BM-InOm neurons are present… only 405 meet the five-synapse threshold”).

(f) LB23 hemilineage: what was that again?

We added text in the manuscript to better define hemilineages. This is described above in response to another Reviewer suggestion.

(g) Line 732: What are ascending neurons?

We had already included a definition of ascending neurons in the second Results section entitled “The BMN connectome”. Since this was not clear to the Reviewers, we expanded on this section. There is now a new paragraph in this same section. This paragraph reads:

“Partners were grouped into five morphological categories—interneurons, descending neurons, ascending neurons, BMNs, and motor neurons—following FlyWire annotations (Dorkenwald et al., 2024). Interneurons were defined as neurons whose soma and all neurites were confined to the brain. Descending neurons were defined as neurons whose somata are located in the CNS and whose neurites extend into the descending tracts toward the ventral nerve cord (VNC). Conversely, ascending neurons were identified as neurons whose neurites enter the brain through the cervical connective and whose somata lie outside the FAFB imaged volume, resulting in only their neurites being visible in the dataset.”

(h) Line 896: What is lineage matching?

We thank the Reviewer for pointing this out. We realized that this sentence did not add clarity and contributed little to the manuscript, so we removed the sentence that used “lineage matching” from the manuscript.

(i) Line 926: The Previous work ... sentence makes no sense to me.

The sentence was reworked and now reads: “The mechanosensory neurons hypothesized from the parallel model that elicit the *Drosophila* grooming sequence were identified in previous work (Eichler et al., 2024; Hampel et al., 2020a, 2017, 2015; Mueller et al., 2019; Seeds et al., 2014; Zhang et al., 2020).”

(j) The FAB-dataset is indeed unique, but the fact that it is repeated several times in your discussion does not ensure understanding of the obviously complex circuit architecture potentially underlying behavior. Please, focus on your discussion strictly and condense your arguments to the specific contribution and outcome of the data in the current manuscript.

In response to this comment, we condensed the opening portion of the Discussion by reducing repetition of background and prior findings, particularly references to earlier BMN work and the uniqueness of the FAFB dataset. We streamlined overlapping sections, mentioned prior hypotheses and results only once, and focused the revised text more directly on the new contributions of this study—namely, the synaptic-resolution organization, somatotopic connectivity, and circuit principles revealed by the BMN connectome.

(k) At some parts of the discussion, it is not clear to me, if you refer to results of the actual study or refer to previous studies (Hampel, Eichler) e.g., 'Our work has shown ...' on line 872.or '...we find ... LB23 neuron elicit antennal grooming....'. or line 909: Our work reveals ......

Sentence a former line 872 was revised and now reads: “While our past and present work together reveal that a subpopulation of LB23 neurons elicits antennal grooming, we also find evidence that other LB23 neurons in the hemilineage elicit additional head grooming movements.”

Sentence at former line 909 was revised and now reads: “Our previous work and the present study reveal that the antennal grooming circuit receives inputs from two different classes of antennal mechanosensory neurons, the BMNs and JONs.”

**Reviewer #3 (Recommendations for the authors):**

All my comments are mostly only for clarity.

(1) It would help readers if the manuscript explicitly stated how a sensory neuron can be postsynaptic - i.e., that BMN axons receive inhibitory inputs in the CNS - since this may not be intuitive to a broader audience.

We appreciate this comment and added the following text to the last paragraph of the first Results section: As expected for sensory afferents, BMNs provide synaptic output to downstream circuits; however, the presence of postsynaptic sites may be less intuitive, and reflects that BMNs can also receive synaptic input onto their central axons within the CNS.

(2) Figure 1 is a helpful context, but since much of it is directly reused from Eichler et al., 2024, it would strengthen the presentation if you clarified what is new here (e.g., the synapse quantification) versus what is recap. In addition, for readers less familiar with EM connectomics, it would be valuable to spell out how bristle neurons are assigned to classes in the absence of bristles themselves in the volume - i.e., that classification rests on stereotyped nerve entry and projection zones, which allow type-level but not single-bristle resolution. Explicitly flagging these methodological boundaries up front would make it clearer what information comes from the current work, what derives from previous reconstructions, and what the limits of resolution are.

We have addressed this recommendation above for a similar suggestion by Reviewer 2 (see above for details). In brief, we inserted an overview of the methodology used to identify BMN types in the FAFB dataset, and we now explicitly state the limitations of this correlative approach. We added a sentence in the first paragraph of the Results section that states, “Because FAFB does not include the head cuticular bristles, individual BMNs could not be linked to single bristles. Therefore, these assignments are necessarily correlative and provide type-level (population) rather than single-bristle resolution.” In addition, we revised the Figure 1 legend to more clearly distinguish panels and reconstructions that were previously reported in Eichler et al. (2024) from synapse quantification and analyses that are new to the present study.

(3) BMNs from neighboring bristle populations converge onto shared partners, while distant BMNs remain segregated - while the overlap was clear, the segregation was not visually clear in the first figure.

We thank the Reviewer for this suggestion. We have addressed this point in our response to a similar comment from Reviewer 2 (see above), where we clarified how overlap versus segregation can be identified in Figure 1 and strengthened the text and figure legends to guide readers to these features without adding clutter to the figure.

(4) The identification of direct BMN → motor neuron synapses is intriguing, but since these inputs make up only a small fraction of motor neuron synapses, it would help if the authors explicitly cautioned readers that these are likely modulatory contributions rather than stand-alone reflex arcs. This would prevent over-interpretation of the sensory-motor link. Similarly with the BMN>BMN connections.

We thank the Reviewer for this suggestion. We revised the Results section “BMN postsynaptic motor neurons” to more explicitly caution that the direct BMN → motor neuron connections are likely modulatory rather than stand-alone reflex arcs, consistent with their small contribution to total motor neuron input. The revised text reads: “However, BMN inputs accounted for only a small fraction of total synapses onto each motor neuron (≦6.28% of total inputs/BMN type, Figure 4 – figure supplement 1, Supplementary file 7), suggesting a modulatory contribution rather than direct sensory-driven motor activation.”

(5) Since the FAFB dataset only includes the brain, it would be helpful to clarify what is meant by "ascending" and "descending" partners in this context - namely that ascending neurons are VNC-derived axons entering the brain, while descending neurons are brain-derived neurons projecting out toward the VNC. Explicitly stating this will prevent confusion, given that all BMNs themselves terminate in the SEZ.

We had already included definitions in the second Results section entitled “The BMN connectome”. Since this was not clear to the Reviewers, we expanded on this section. There is now a new paragraph in this same section. This paragraph reads: Partners were grouped into five morphological categories—interneurons, descending neurons, ascending neurons, BMNs, and motor neurons—following FlyWire annotations (Dorkenwald et al., 2024). Interneurons were defined as neurons whose soma and all neurites were confined to the brain. Descending neurons were defined as neurons whose somata are located in the CNS and whose neurites extend into the descending tracts toward the ventral nerve cord (VNC). Conversely, ascending neurons were identified as neurons whose neurites enter the brain through the cervical connective and whose somata lie outside the FAFB imaged volume, resulting in only their neurites being visible in the dataset.

(6) In the section titled "BMN synaptic partners in the CNS: ascending, descending, and interneurons", the balance of explanation is skewed toward presynaptic input to BMNs. It would strengthen clarity if you expanded equally on the postsynaptic side (i.e., BMN outputs) or explicitly signposted why the focus here is on inputs. That way, readers won't be left wondering whether outputs are less important or just deferred to later figures.

We have revised the section that was previously skewed toward presynaptic BMNs. This section also addresses some confusion about interpreting Figure 3, from a critique from Reviewer 2. The section now reads: “Postsynaptic connections were predominantly interneurons (56%), with significant contributions from descending (28%) and ascending (16%) neurons (Figure 5D, F,H,J). Interneurons are more numerous as distinct partner neurons, whereas descending neurons receive a larger fraction of BMN output synapses across BMN types (Figure 3A, B). Thus, descending neurons are fewer in number but tend to receive more BMN synapses per neuron on average, while interneurons are more numerous but often receive fewer synapses per neuron. Together, these partner categories underscore the strong integration of BMNs with local brain circuitry (interneurons), and with pathways linking the brain and ventral nerve cord (VNC), through ascending neurons that provide VNC-derived synaptic input and descending neurons that carry BMN output toward the VNC.”

(7) The network diagrams in Figure 9 convey clustering, but a complementary heatmap of BMN type × partner connectivity could highlight the parallel organization more clearly. This would make the block-like separation of dorsal, ventral, and posterior subnetworks more immediately apparent, reinforcing the conclusion of parallel somatotopy-based processing. This section would also benefit from drawing the functional message more explicitly: that BMNs form largely independent, somatotopically aligned pathways with regional overlap, supporting the idea of parallel grooming circuits. Right now, the text reads as a connectivity catalog, and the key concept of parallel regional architecture risks being underemphasized.

We agree that connectivity matrices provide a clear and accessible representation of these data. We have moved the cosine-similarity connectivity matrices previously shown in Figure 9 – figure supplement 1 into the main manuscript, where they now appear as Figure 9. These matrices depict similarity among BMN postsynaptic partners. For this reason, we have retained the original network-graph representation (now Figure 10) to display the full pre- and postsynaptic connectome structure.

Based on the Reviewer’s suggestion to clearly state the key concepts of the parallel architecture, we added a sentence to the end of the Results section entitled: Somatotopy-based connectivity among BMN synaptic partners in the CNS. That text reads: “Thus, the BMNs form largely independent, somatotopically aligned pathways with regional overlap, supporting the idea of parallel grooming circuits.”

(8) It would help if the manuscript if the authors explained more explicitly the somatotopy logic (that reciprocal inhibition preserves local head regions, ensuring that suppression and gain control act locally) more clearly. At present, the narrative is buried in network-graph detail - a heatmap or simple region-level summary would make this organizational principle much clearer to readers.

We thank the Reviewer for this suggestion. To make the somatotopy logic of pre/post feedback inhibition clearer and less buried in network-graph detail, we revised the text in this Results section to more explicitly distinguish (i) reciprocal, head-region–localized inhibitory loops that could support local gain control from (ii) non-reciprocal cross-type inhibitory pathways that could contribute to heterotypic suppression between head regions. In addition, we modified the figure to more clearly convey somatotopy by adding text on the plot and updating the legend to state: “Bold text indicates the general head location of BMNs on the plot, revealing somatotopy-based connectivity with pre/post neurons (i.e. ventral, dorsal, posterior, and the ventral/dorsal transition).”

(9) Please adjust the section title, "LB23 hemilineage member neurons elicit aimed head grooming movements" to avoid implying new functional experiments. For example:

(a) "LB23 neurons include previously defined antennal grooming command neurons" or

(b) "LB23 hemilineage anatomically corresponds to grooming-related neurons".

This would make it clear that the contribution here is anatomical linkage, not fresh functional data.

We changed the section title to the Reviewer-suggested title b: LB23 hemilineage anatomically corresponds to grooming-related neurons

(10) The current network graphs in Figure 13B are not very intuitive - it is hard to visually extract the somatotopy. A connectivity heatmap or matrix (BMN types on one axis, LB23 neurons or subgroups on the other, with synapse strength as colour) would make the block-like, region-specific mapping immediately clear. A coarse-grained version (e.g., dorsal/ventral/posterior BMNs vs LB23 subgroups) could further highlight the parallel, somatotopically organized pathways. This would better support the central claim of Figure 13 than the current spring-layout graphs. Figure 13F does this for BMN inputs onto aBN2 neurons. (But it is presented only in binary form; could the authors not add a graded colour scale proportional to synapse number?)

The binary form was necessary because the results are from different sources (i.e. Catmaid versus flywire synapse counts) with different synapse numbers.

We modified the Figure 13B to more clearly convey somatotopy by adding text on the plot and updating the legend to state: “Bold text indicates the general head location of BMNs on the plot, revealing

somatotopy-based connectivity with LB23 neurons (i.e. ventral, dorsal, and posterior head).” We hope that this modification satisfies the Reviewer.
